# Ethyl Acetate Fraction of *Amomum villosum* var. *xanthioides* Attenuates Hepatic Endoplasmic Reticulum Stress-Induced Non-Alcoholic Steatohepatitis via Improvement of Antioxidant Capacities

**DOI:** 10.3390/antiox10070998

**Published:** 2021-06-23

**Authors:** Jung-Hyo Cho, Jong-Suk Lee, Hyeong-Geug Kim, Hye Won Lee, Zhigang Fang, Hyeok-Hee Kwon, Dong Woon Kim, Chang-Min Lee, Jin-Woo Jeong

**Affiliations:** 1 Department of Biochemistry and Molecular Biology, School of Medicine, Indiana University, Indianapolis, IN 46202, USA; choajoa@dju.ac.kr (J.-H.C.); kim12@iu.edu (H.-G.K.); 2 Department of East & West Cancer Center, Daejeon Korean Medicine Hospital of Daejeon University, 75, Daedeok-daero 176, Seo-gu, Daejeon 35235, Korea; 3 Biocenter, Gyeonggido Business & Science Accelerator (GBSA), Suwon 16229, Korea; leejs@gbsa.or.kr; 4 Herbal Medicine Research Division, Korea Institute of Oriental Medicine, Daejeon 34054, Korea; hwlee@kiom.re.kr; 5 Department of General Surgery, Guangzhou First People’s Hospital, School of Medicine, South China University of Technology, Guangzhou 510180, China; eyfzg@scut.edu.cn; 6 Department of Anatomy and Cell Biology, Brain Research Institute, Chungnam National University College of Medicine, Daejeon 35015, Korea; kara00124@gmail.com (H.-H.K.); visnu528@gmail.com (D.W.K.); 7 Nakdonggang National Institute of Biological Resources, 137, Donam 2-gil, Sangju-si 37242, Gyeongsang-buk-do, Korea; ing2020@nnibr.re.kr

**Keywords:** *Amomum villosum* var. *xanthioides* (Wall. ex Baker) T.L.Wu & S.J.Chen, endoplasmic stress, non-alcoholic steatohepatitis, oxidative stress, metallothionein

## Abstract

Non-alcoholic fatty liver disease (NAFLD), including non-alcoholic steatohepatitis (NASH), affects 25% of the global population. Despite the prevalence of NAFLD worldwide, effective therapeutics are currently lacking. *Amomum villosum* var. *xanthioides* (Wall. ex Baker) T.L.Wu & S.J.Chen (AX) is a medicinal herb traditionally used for treating digestive tract disorders in countries across Asia. We aimed to examine the pharmacological effects of the ethyl acetate fraction of AX (AXEF) against tunicamycin (TM)-induced ER stress in a NASH mouse model using C57/BL6J male mice. Following TM injections (2 mg/kg), the mice were orally administrated AXEF (12.5, 25, or 50 mg/kg), silymarin (50 mg/kg), or distilled water daily for 5 days, and the outcomes for fatty liver, inflammation, and oxidative stress were measured in serum or liver tissue levels. AXEF drastically attenuated hepatic ER stress-induced NASH as indicated by decreases in lipid droplet accumulations, serum liver enzymes, hepatic inflammations, and cell death signals in the hepatic tissue and/or serum levels. Interestingly, AXEF showed potent antioxidant effects by quenching reactive oxidative stress and its final product lipid peroxide in the hepatic tissue, specifically an increase in metallothionein (MT). To confirm the underlying actions of AXEF, we observed that AXEF increases MT1 gene promoter activities in the physiological levels. Collectively, AXEF showed antioxidant properties on TM-induced ER stress in a NASH mice model through the improvement of MTs.

## 1. Introduction

As a representative metabolic disease in the liver tissue, non-alcoholic fatty liver disease (NAFLD) is related to a spectrum of liver diseases including simple fatty liver, steatohepatitis, liver fibrosis, and hepatocellular carcinoma, which is an end stage of liver disease [1,2]. NAFLD is generally characterized by excessive lipid accumulation in the liver (over 5%) and is accompanied by lipid metabolism dysregulation [3]. Consequently, it excessively progresses secondary pathological conditions such as hepatic inflammation and liver tissue oxidation stress [4,5]. Non-alcoholic steatohepatitis (NASH) is an advanced type of NAFLD characterized by fatty liver with inflammation throughout the liver tissue. The prevalence of both types of liver disease is approximately 25% worldwide and, unfortunately, there have been no effective therapeutic strategies suggested until recently [6,7,8,9].

The endoplasmic reticulum (ER), a major intracellular organelle involved in lipid metabolism in hepatocytes, plays a crucial role in fatty liver and lipid accumulations in the liver [10,11]. Hepatic ER stress contributes to severe hepatic injury through mechanisms including the activation of cell death signals [12], disturbance of calcium ion homeostasis [13], dysregulation of autophagic flux [14], and hepatic inflammation [15]. These pathological statuses are deeply associated with hepatic oxidative stress [16,17,18]. Excessive lipid droplet accumulation generates reactive oxygen species (ROS) and induces lipid peroxidation, both of which are deeply related to the hepatic inflammatory reactions [19]. Altogether, this suggests that to relieve or prevent oxidative stress in the liver would be an appropriate strategy to treat NASH [20,21].

*Amomum villosum* var. *xanthioides* (Wall. ex Baker) T.L.Wu & S.J.Chen, also known as Amomi Fructus, is a species of *Amomum villosum* Lour (AX) in the genus of *Amomum* and belongs to *Zingiberaceae* family [22,23,24,25]. AX has been traditionally used to treat patients with digestive disorders by enhancing stomach motility with the removal of moisture, warming the stomach and stopping diarrhea, and enhancing the energy for maternities [26,27,28,29]. Previous studies showed the pharmacological properties of AX on the chronic liver disease models with variety fractionized extracts well [30,31,32]. Among the various types of fractions, the ethyl acetate fraction of AX (AXEF) specifically showed anti-fibrotic effects [30,31]. Additionally, a recent study evidenced AXEF effects on the 8 weeks feeding in an HFD-induced NAFLD mice model [33]. Although previous studies have established the pharmacological effects of AXEF on liver diseases, no studies have explored the pathophysiological features and underlying mechanisms of AXEF on the hepatic ER stress-related NASH status.

Thus, in the present study, we investigated the pharmacological effects of AFEX and its underlying mechanisms using hepatic ER stress-provoked NASH conditions in a tunicamycin (TM)-injected mice model by focusing on hepatic metallothionein (MT).

## 2. Materials and Methods

### 2.1. Preparations of AXEF and Finger Printing Analysis

AX was obtained from Dong-yang-dang Oriental Herbal Medicinal Market (Pung-gi, Kyuong-Book, South Korea). Dried AX was washed using tap water twice, then rinsed with distilled water (DW) twice. These materials were completely dried in a dry oven (60 °C) overnight and prepared using a grinder-mixer. AX was extracted in absolute methanol for 3 days with shaking (every 24 h thrice at RT). The methanolic fraction was obtained by adding DW (9:1, 9 for methanol and 1 for DW). The residue (in particular the DW fraction) was collected and extracted with acetone, similar to the methanol fraction by adding DW. Then, the residue was extracted using ethyl acetate and a further fractionized step was completed to finally obtain lipolyzed AXEF samples (final yield of 0.2%, Supplementary Figure S1).

Next, we investigated the chemical composition characteristics of AXEF using ultra-high-performance liquid chromatography–tandem mass spectrometry (UHPLC–MS/MS). Briefly, a 5-milligram aliquot of the AXEF samples were dissolved in 1 mL of 90% methanol, then filtered (0.45 μm). Then, 10 μL of the AXEF sample solutions were then subjected to UHPLC–MS using an LTQ Orbitrap XL linear ion-trap MS system (Thermo Scientific Co., San Jose, CA, USA) that was equipped with an electrospray ionization source. Separation of each AXEF sample was performed on an Accela UHPLC system with an Acquity BEH C18 column (1.7 μm, 100 × 2.1 mm; Waters, Milford, MA, USA). The column was eluted at a flow rate of 0.4 mL/min using DW (in 0.1% formic acid) and acetonitrile (in 0.1% formic acid), which were applied to mobile phases as A and B, respectively; mobile phase compositions were followed as the following gradient modes: 0–1 min, 0–1% B in A; 1–7 min, 1–100% B in A; 7–10 min, 100–1% B in A (linear gradient). We obtained chemical compositional analysis using a photodiode array at 200–600 nm, and full-scan mass spectra were acquired at 150–1500 *m*/*z* in both positive and negative modes. An Orbitrap analyzer was used for high-resolution mass data acquisition with a mass resolving power of 30,000 FWHM at 400 *m*/*z*. Tandem mass (MS/MS) spectra were acquired in data-dependent mode by collision-induced dissociation. From the chemical analysis results, we confirmed that most of the compounds in AXEF were from phenolic acid derived- and flavonoid derived-compounds, which are well known to the various pharmacological properties of numerous diseases.

### 2.2. Animals Experiment

We purchased 8-week-old male C57/BL6 mice from Deahan biolink (Cheongju, South Korea). Animals were acclimated to the temperature (22 ± 2 °C) and humidity (55 ± 5%) of controlled rooms with 12 h light and dark cycles for seven days. After acclimation, the mice were orally administrated AXEF (0, 12.5, 25, or 50 mg/kg) or silymarin (50 mg/kg) for five consecutive days, then the mice were intraperitoneally (i.p.) injected with saline or TM (2 mg/kg) 6 h after the fifth drug ingestion. All mice were sacrificed following an avertin injection (250 mg/kg, i.p.) of anesthesia, 48 h post TM injection. In the present study, we applied silymarin, which is well known as ‘Milk Thistle’, based on the accumulated protective effects on various liver diseases [34,35,36,37].

The liver tissues were removed, fixed using 10% neutral formalin, and stored at −70 °C for further use. For liver enzymes, whole blood samples were collected from the abdominal vein. All animal procedures performed in the current study were approved by the Institutional Animal Care and Use Committee of Chung-Nam National University Hospital followed by National Institutes of Health guidelines for the care and use of laboratory animals (CNUH-019-A0033-1).

### 2.3. Hepatic Triglyceride (TG) Measurement

Hepatic lipids were prepared from liver tissues via the addition of a chloroform-methanol mixture (2:1) extraction method, as described previously [38]. The hepatic TG level was measured using Wako L-type TG assay kits (FUJIFILM Wako Diagnostics, Richmond, VA, USA).

### 2.4. Liver Enzyme Examinations

Whole blood was collected from the abdominal aorta on the final date of the experiment. Serum samples were obtained using centrifugation (5000× *g*, 30 min) after blood clotting for 1 h at RT. Serum levels of liver enzymes, including aspartate transaminase (AST), alanine transaminase (ALT), and lactate dehydrogenase (LDH), were measured using commercially available kits (Thermo Scientific™ Data Trol™ Normal Control Serum for AST; Pointe Scientific, #A7526150 for ALT; Invitrogen™ CyQUANT™ LDH Cytotoxicity Assay for LDH, respectively).

### 2.5. Histopathological Analysis and ROS Analysis in the Mice Liver Tissue

The liver tissues were fixed in 10% buffered neutral formalin and embedded in a low-melting-point paraffin, following normal protocol. We prepared tissue sections 4 µm thick and applied hematoxylin and eosin (H&E) staining to perform pathological examinations. To detect lipid droplet accumulation in the liver tissue, we performed Oil Red O staining by preparation of an optimal cutting temperature (OCT) compound. After gradient sucrose incubation (10 to 30% per overnight) for cryoprotection, samples were frozen, sectioned (8 μm thick), then stained.

To examine the accumulation of superoxide anions or H_2_O_2_, we applied dihydroethidine hydrochloride (DHE, 2 µM) or 5-[and-6]-chloromethyl-2′,7′-dichlorodihydrofluorescein diacetate (CM-H2DCFDA; Invitrogen, 5 µM) to the frozen liver tissues. The tissues were incubated at 37 °C for 30 min in a humidity chamber. The positive signals of fluorescence were detected using fluorescent microscopy [39].

### 2.6. Analysis of Immunohistochemistry (IHC) and Terminal Deoxynucleotidyl Transferase dUTP Nick End Labeling (TUNEL) Analysis

We applied IHC analysis against 4-hydroyneanel (4HNE) to detect lipid peroxidation in the liver tissue. Liver sections (4 µm thick) were prepared using deparaffinizing and rehydrating progressions and microwaving in an antigen retrieval buffer (sodium citrate 10 mM, pH 6.0) for 5 min. After cooling and washing, we added 3% H_2_O_2_ in methanol to remove erythrocytes. Then, we incubated the blocking buffer for 1 h at RT and applied anti-mouse-4-HNE (R&D system, MA, Cat. #MAB3249, 1:200) overnight at 4 °C. The next day, after washing, we applied a secondary antibody and developed the positive signals using 3,3′-Diaminobenzidine (DAB) after amplifying the HRP (ImmPACT^®^ DAB Substrate, Cat. #SK-4105). We used ICH, a commercially available product, as the reagent (VECTASTAIN^®^ ABC Kits, Burlingame, CA, USA). A TUNEL (terminal deoxynucleotidyl transferase dUTP nick end labeling) assay was performed using a commercial kit (Sigma-Aldrich Chemical Co., St. Louis, MO, USA).

### 2.7. Cell Culture and In Vitro NASH Model

Human liver cell line, Huh7 cells, were cultured in Dulbecco’s modified eagle’s medium (DMEM) with 10% fetal bovine serum (FBS), 2 mM glutamine, 100 U/mL penicillin, and 100 µg/mL streptomycin (Thermo Fisher Scientific, Waltham, MA, USA) at 37 °C under the humidified condition of 5% CO_2_ supplement. An in vitro NASH model, which is provoked by ER stress, was complete by treatment with TM (5 µM) for 16 h incubation. To investigate the pharmacological properties of AXEF (5 and 10 µg/mL), we pre-treated with the drug 4 h before TM treatment. To decide the AXEF dosages, we referred to a previous study of AXEF using a cell-based liver fibrosis model [30], after a cytotoxic assay. To obtain the effects of AXEF against a pharmacological ER stress-induced NASH cell mode, we performed Oil Red O staining, a TUNEL assay (Sigma Millipore), LIVE/DEAD cell analysis (Thermo Fisher Scientific, Waltham, MA, USA), and immunocytochemistry analysis for IHC or IF methods targeting 4-HNE (1:200) or pH2Ax (1:200), respectively.

### 2.8. Biochemical Analysis

We examined biochemistry analysis, including ROS, lipid peroxidation, and the total glutathione (GSH) contents in the hepatic tissue and cellular protein levels. For hepatic protein extract, frozen hepatic tissues were homogenized in a lysis buffer, which contains 50 mM 4-(2-hydroxyethyl)-1-piperazine ethane sulfonic acid with a pH 7.5, 150 mM NaCl, 10% glycerol, 1% Triton X-100, 1.5 mM MgCl2, 1 mM ethylene glycol tetra-acetic acid, 10 mM sodium pyrophosphate, 100 mM sodium fluoride, freshly added 100 μM sodium vanadate, and 1 mM phenyl-methyl-sulfonyl fluoride (PMSF) with a complete protease inhibitor cocktail tablet (Roche, Indianapolis, IN). We measured the protein concentration of each of the lysate samples using the bicinchoninic acid (BCA) method (Pierce™ BCA Protein Assay Kit, Thermo Fisher). Cellular protein was prepared using a Nonidet P-40 (NP-40) lysis buffer, which contains 1% NP-40, 20 mM Tris with a pH 7.4, 137 mM NaCl, 2 mM ethylene diamine tetra-acetic acid (EDTA), 10% glycerol, and 1 mM PMSF with a complete protease inhibitor cocktail tablet. Biochemical assay, including hydrogen peroxide (H_2_O_2_), lipid peroxidation, and total GSH contents, was performed using a commercially supplied kit (Amplex™ Red Hydrogen Peroxide/Peroxidase Assay Kit, Invitrogen™, Thermo Fisher Scientific, Cat.#: A22188 for ROS; Lipid Peroxidation (measured the contents of malondialdehyde; MDA) Colorimetric/Fluorometric Assay Kit, BioVision, Cat. #: K739 for lipid peroxidation; Glutathione Assay Kit, Cat. #: CS0260, Sigma-Aldrich, respectively).

### 2.9. Western Blot Analysis

Hepatic and cellular protein samples, which were whole protein lysates with equal amounts (50 µg per each sample), were separated using sodium dodecyl sulfate (SDS)–polyacrylamide gel electrophoresis, then SDS gels were transferred to nitrocellulose (NC) membranes for Western blot analysis. Membranes were incubated in a blocking buffer of 5% BSA in TBST and applied to the specific primary antibodies and incubated at 4 °C for overnight. Then, we applied a secondary antibody and developed chemiluminescence (Thermo Fisher Scientific, Waltham, MA, USA) signals for detecting the positive signals. The antibody conditions are available in Supplementary Table S1.

### 2.10. Real-Time Reverse Transcription-Polymerase Chain Reaction (RT-PCR)

Total RNAs from tissues and cells were isolated using a TRI reagent (Sigma, St. Louis, MO, USA). A total of 1 μg of RNA was used for cDNA synthesis using a reverse transcription kit (Applied Biosystems, Foster City, CA, USA), then applied to the synthesis of cDNA, which was used for real-time qPCR analysis using a SYBR Green qPCR kit (Applied Biosystems, Foster City, CA). The qPCR reactions were performed on a RealPlex2 thermal cycler (Eppendorf, Hauppauge, NY, USA) and cDNA amounts were calculated according to a standard curve using the reference gene β-actin. Primer sequences are described in Supplementary Table S2.

### 2.11. Measurement of Caspase-3/7 Activities

We measured caspase-3 and 7 activities using a commercially available kit (Caspase-Glo^®^ 3/7 Assay System, Promega, Madison, WI, USA), according to the manufacture’s standard protocol, using a 96-well micro plate and the 200 µL method. For hepatic protein, we transferred 100 µL of hepatic protein lysates to a 96-well microplate, then added a total of 100 µL of the mixed reagent (Caspase-Glo^®^ 3/7 Substrate and Caspase-Glo^®^ Buffer, Promega, Madison, WI, USA) to each well. After shaking the plate, it was incubated for 3 h at RT.

For in vitro analysis, Huh7 cells (1 × 10^4^ cells/well) were cultured with 10% FBS-supplemented DMEM in a cell incubator. After being left overnight, cells were changed to a fresh non-FBS containing DMEM with AXEF (5 or 10 µg/mL) or silymarin (50 µg/mL) for 4 h before TM (5 µg/mL) treatment. Then, cells were further incubated for 16 h. Then, a total of 100 µL of the mixed reagent was added to the media-containing wells and the plate was put on a shaker for 30 s (500 rpm). After shaking, the plate was incubated at RT for 2 h, then signals were detected using a luminometer. The values were normalized by a control group and expressed fold changes. The blank, negative and positive wells were all measured according to the manufacturer’s protocol.

### 2.12. Gene Promoter Reporter Assay

We analyzed luciferase using mouse Mt1 and Mt2 gene promoters with a pGL4.10 luciferase firefly reporter system by transfection of HEK 293T cells. Briefly, HEK 293T cells (5 × 10^5^/wells) were seeded to a 24-well plate with 10% FBS-supplemented DMEM overnight in a cell incubator. Each plasmid for MT1-pcDNA, MT2-PcDNA, MTF1, GFP, and Renilla were co-transfected (a total 1 µg). AXEF 10 µg was treated without the transfection conditions of metal regulatory transcription factor 1 (MTF1) and GFP. After a 24-h incubation, luciferase assay was performed using a Dual-Luciferase^®^ Reporter Assay System (Promega). The substrate was detected using a luminometer. Data were analyzed by normalization of an internal control for Renilla (SV-40) as the luciferase reporter, to previously described [7].

### 2.13. Statistical Analysis

All results, expressed as means ± Standard Deviations (SD), were analyzed using a one-way analysis of variance (ANOVA) followed by the Tukey’s HSD test using Prism ver. 9.1 (GraphPad, CA, USA). The acceptable level of significance was established at *p* < 0.05.

## 3. Results

### 3.1. AXEF Attenuated ER Stress-Mediated NASH of TM Injection Mice Model

**Figure 1:**
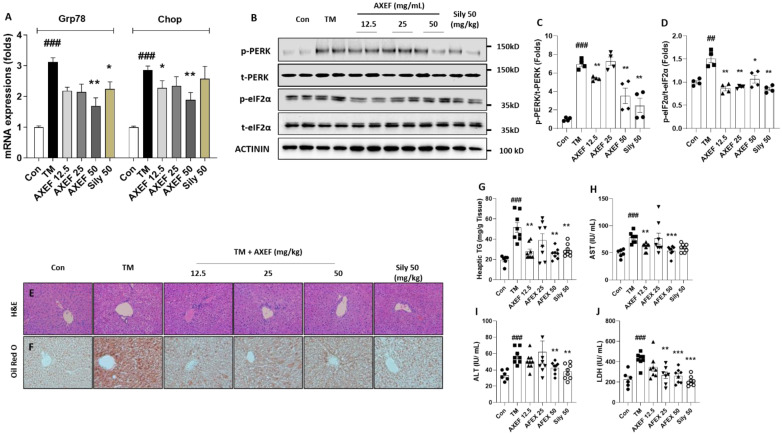
Effects of AXEF on the ER stress and NASH by TM injection in a mice model. (**A**) Hepatic mRNA expression levels of Hspa5 and Ddit3. (**B**) Western blot analysis of ER stress molecules of p-PERK, t-PERK, p-eIF2α, and t-eIF2α in the hepatic protein lysates. (**C**) and (**D**) Protein intensity of ER stress related proteins for p-PERK, t-PERK, p-eIF2α, and t-eIF2α. (**E**) Hepatic H&E staining and (**F**) Oil Red O staining. (**G**) Hepatic tissue levels of TG and liver enzymes in the serum levels including (**H**) AST, (**I**) ALT, and (**J**) LDH, respectively. Data are expressed as the mean ± SD (*n* = 6–8 for each group for PCR and hepatic TG, AST, ALT, and LDH; *n* = 4 for Western blot analysis, respectively). ## *p* < 0.01; ### *p* < 0.001 for the Con vs. TM, * *p* < 0.05; ** *p* < 0.01; *** *p* < 0.001 for TM vs. AXEF or Sily 50. Images were captured using light microscopy at 100 or 200× magnification.

We observed that hepatic ER stress was induced by single injection of TM by the performance of qPCR and Western blot analysis. Hepatic mRNA expression levels of Grp78/BiP and Didit3/Chop were significantly higher, approximately 3.1- and 2.9-fold that of the control group, respectively (*p* < 0.001 for both, Figure 1A). Additionally, the ER stress-related proteins, including phosphor-PERK and phspho-eIF2α, were drastically increased compared with the control group (*p* < 0.001 or 0.01 for Figure 1B–D). These ER stress markers in hepatic tissue levels were significantly normalized by the AXEF pre-administration groups (Figure 1A–D, *p* < 0.05, 0.01, or 0.01 for qPCR analysis and Western blot analysis).

The H&E staining of the mice liver tissue sections after the TM injection showed that the hepatocellular structure was altered by hepatocyte ballooning and inflammation. These statuses were apparently evidenced NASH phenotypes, such as lipid accumulations in the hepatocytes and inflamed cells infiltrations (Figure 1E). In addition, Oil Red O staining was also featured by remarkable increases in the lipid droplets’ accumulation in the TM group (Figure 1E). Pre-administration with AXEF drastically ameliorated these NASH conditions, compared with the TM group.

The hepatic TG levels in the TM group increased around 2.6-fold compared with the control group (*p* < 0.001 for Figure 1G), which were also significantly reduced by AXEF pre-administration (Figure 1G). Regarding lipogenesis, the related genes, including Plin2, Dgat2, Hmgr2, and Acca2, were significantly up-regulated around 2.3-, 2.7-, 2.6., and 2.8-fold compared to that of control group, and these abnormalities were significantly normalized by AXEF (Supplementary Figure S2, *p* < 0.05 or 0.01).

Along with the above results, serum biochemistries including AST, ALT and LDH were considerably increased due to hepatic ER stress by 1.4-, 1.7, and 2.1-fold, respectively, compared with the control group (*p* < 0.001 for Figure 1H–J), and AXEF significantly ameliorated the serum liver enzyme levels (*p* < 0.05, 0.01, or 0.001 in Figure 1H–J).

Pre-administration with 50 mg/kg silymarin, as a positive control group, also showed similar properties of AXEF.

### 3.2. AXEF Improved Hepatic ER Stress-Induced Inflammation

**Figure 2:**
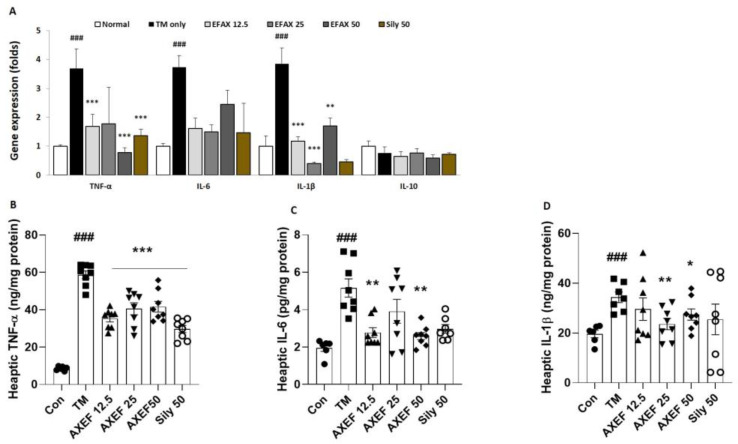
Anti-inflammatory properties of AXEF on the ER stress-induced NASH mice model. (**A**) Hepatic mRNA expression levels of inflammatory response related cytokines, including TNF-α, IL-1β, IL-6, and IL-10, respectively. (**B**–**D**) Hepatic protein levels of pro-inflammatory cytokines including TNF-α, IL-1β, and IL-6, respectively. Data are expressed as the mean ± SD (*n* = 6–8 for each group). ### *p* < 0.001 for the Con vs. TM, * *p* < 0.05; ** *p* < 0.01; *** *p* < 0.001 for TM vs. AXEF or Sily 50.

**Figure 3:**
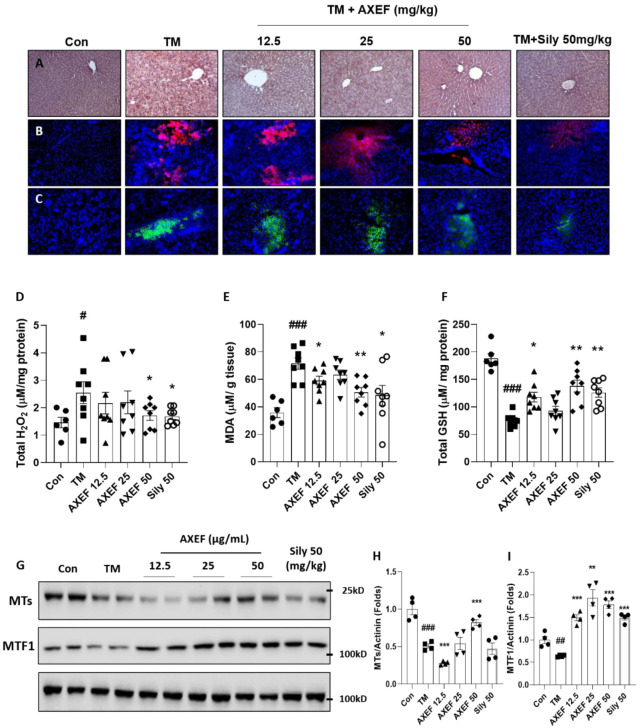
Antioxidant effects of AXEF on the ER stress-induced NASH mice model via improvement of hepatic MTs. (**A**) Immunohistochemistry analysis against 4-HNE, (**B**) fluorescence quenching analysis of DHE (red fluorescence), and (**C**) DCF-DA (green fluorescence). DAPI was used to counter stain the nucleus (Blue fluorescence). Hepatic protein levels of (**D**) H_2_O_2_, (**E**) MDA, and (**F**) GSH were measured. (**G**) Western blot analysis of hepatic protein levels of MT and MTF1. (**H**,**I**) Protein intensity levels of hepatic MT (**H**) and MTF1 (**I**)**.** Data are expressed as the mean ± SD (*n* = 3–4 for IHC analysis and hepatic ROS quenching analysis; *n* = 6–8 for each group for hepatic H_2_O_2_, MDA, and total GSH levels; *n* = 4 for Western blot analysis, respectively). # *p* < 0.05; ## *p* < 0.01; ### *p* < 0.001 for the Con vs. TM, * *p* < 0.05; ** *p* < 0.01; *** *p* < 0.001 for TM vs. AXEF or Sily 50. Images were captured using light microscopy (200× magnification) or fluorescence filtered microscopy (200× magnification).

Our qPCR analysis results for pro-inflammatory cytokines including TNF-α, IL-6, and IL-1β exhibited significant up-regulations of approximately 3.6-, 3.7-, and 3.8-fold, respectively, compared to that of the control group (*p* < 0.001), whereas the IL-10 of the mRNA expression level showed a tendency for down-regulation, around 0.7-fold compared with the control group (Figure 2A, *p* > 0.05). The hepatic protein levels of the above pro-inflammatory cytokines were also similar to the qPCR results, which were significantly increased by 1.6-, 1.9-, and 3.3-fold compared to the control group by order of TNF-α, IL-6, and IL-1β, respectively (*p* < 0.001 for Figure 3B). Pre-administration with AXEF worked to significantly normalize the above abnormal alterations in both mRNA expression and hepatic protein levels (*p* < 0.05, *p* < 0.001, or *p* < 0.001 for Figure 2A,B).

Regarding hepatic inflammation, the positive control group, which was pre-administered with 50 mg/kg silymarin, also showed similar properties of AXEF.

### 3.3. AXEF Exerted to Reduce Hepatic ER Stress-Mediated Oxidation

To validate that the AXEF effects were elucidated in antioxidative properties, we performed IHC against 4-HNE, which is a marker of lipid peroxidation known as a final product of oxidative stress. In the TM group, the positive signals of 4-HNE were considerably increased compared with the control group (Part of brown color, Figure 3A). We further explored the fluorescence analysis for free radicals such as DHE (for superoxide radicals with red fluorescence for positive signal) and DCF-DA (for hydrogen peroxide with green fluorescence for positive signal), which were also associated with the TM-injection-induced hepatic ER stress NASH model in the present study. As we expected, both of the fluorescence signals were drastically enhanced in the TM group compared with the control group (Figure 3B,C). Including IHC analysis against 4-HNE, the free radical fluorescence analyses were considerably weakened by the AXEF pre-administrations (Figure 3A–C).

In accordance with the above pharmacological properties of AXEF, it improved the abnormal elevations of both H_2_O_2_ (1.7-fold higher, *p* < 0.05 for Figure 3D) and MDA (2.0-fold higher, *p* < 0.001 for Figure 3E) and hepatic protein levels (*p* < 0.05 for H_2_O_2_ and *p* < 0.05 or 0.01 for MDA). Additionally, AXEF also significantly led to the recovery of hepatic GSH deteriorations (deteriorations of 0.4-fold, *p* < 0.001 for Figure 3F) due to the TM injection compared with the control group (*p* < 0.05 or 0.01).

Silymarin also exhibited strong antioxidant capacities well by relieving the hepatic tissue oxidation and increasing the hepatic GSH levels.

### 3.4. AXEF Exerted to Improve Hepatic MTs

To verify that the hepatic MT protein levels were implicated in the ER stress by TM injection mice model, we performed Western blot analysis. The hepatic MTs and their transcription factor MTF1 in the hepatic protein levels were significantly decreased in the TM group compared with the control group (*p* < 0.01 or 0.001 for Figure 3G–I); however, pre-administration with AXEF significantly recovered the deterioration of both hepatic protein levels of MT and MTF1 (*p* < 0.01 or 0.001 for Figure 3G–I).

Silymarin also significantly increased the hepatic MTF1 protein levels compared with slight increases in the TM group but did not affect the hepatic MT protein levels.

### 3.5. Anti-Apoptotic Effects of AXEF on the ER Stress-Induced NASH Mice Model

**Figure 4:**
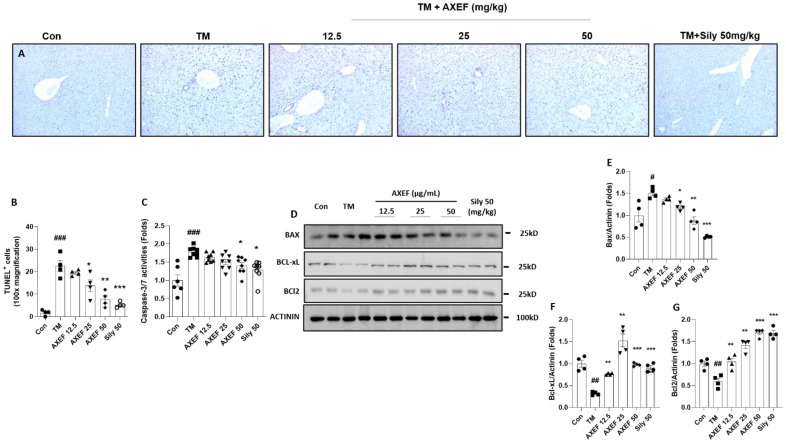
Anti-apoptotic effects of AXEF on the ER stress-induced NASH mouse model. (**A**) TUNEL assay and (**B**) quantitative analysis of TUNEL positive signal through the liver tissue. (**C**) Hepatic protein levels of caspase-3/7 activities. (**D**) Western blot analysis of apoptosis-related proteins BAX, BcL-xL, and BcL-2 in the hepatic protein lysates. (**E**–**G**) Quantitative analyses of Western blot. Data are expressed as the mean ± SD (*n* = 4 for TUNEL assay and Western blot analysis; *n* = 6–8 for each group for caspase-3/7 activities). # *p* < 0.05; ## *p* < 0.01; ### *p* < 0.001 for the Con vs. TM, * *p* < 0.05; ** *p* < 0.01; *** *p* < 0.001 for TM vs. AXEF or Sily 50. Images were captured using light microscopy (100× magnification).

As we expected, hepatocyte death due to ER stress occurred in the TM group compared with the control group (*p* < 0.001), which was evidenced by TUNEL positive signals; however, pre-administration with AXEF significantly attenuated them compared with the TM group (*p* < 0.05 or 0.01 for Figure 4A,B). The caspase-3/7 activities in the hepatic protein levels of the TM group were significantly increased by the TM injection, whereas these abnormal increases were significantly decreased by AXEF pre-administration (Figure 4C, *p* < 0.05).

Western blot analysis results showed that a pro-apoptosis related protein, especially BAX, was notably increased in the TM group compared with the control group (*p* < 0.05), but these signals significantly decreased in the AXEF group (*p* < 0.05 or 0.01 for Figure 4D,E). BcL-xL and BcL-2, which are known as anti-apoptosis related proteins, were significantly decreased by the TM injection (*p* < 0.01); however, pre-administration with AXEF recovered those deteriorations during the ER stress response in the hepatic tissue (*p* < 0.01 or 0.001 for Figure 4D,F,G, respectively).

A positive drug in the current manuscript, silymarin 50 mg/kg also showed anti-apoptosis effects on the TUNEL assay, caspase-3/7 activities, decreases in the haptic protein levels of BAX, and increases in both BcL-xL and BcL-2, respectively.

### 3.6. Antioxidant Effects of AXEF on TM-Induced Cellular ER Stress of NASH Model via Improvement of MTs in Huh7 Cells

**Figure 5:**
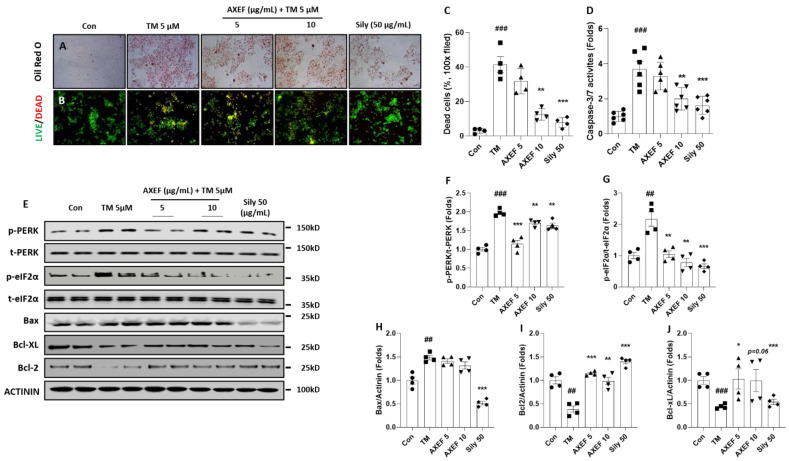
Effects of AXEF on the ER stress-induced in vitro NASH model of cellular damage. (**A**) Oil Red O staining of Huh7 cells of TM-induced (5 µM) ER stress in vitro NASH model using treatment and (**B**) LIVE/DEAD cell assays. (**C**) Quantitative analysis of LIVE/DEAD cell assay. (**D**) Cellular levels of caspase-3/7 activity. (**E**) Western blot analysis of ER stress and apoptosis-related molecules from Huh7 cell lysate. (**F**–**J**) Data are expressed as the mean ± SD (*n* = 4 for Oil Red O staining; *n* = 6–8 for caspase-3/7 activities). ## *p* < 0.01; ### *p* < 0.001 for the Con vs. TM, * *p* < 0.05; ** *p* < 0.01; *** *p* < 0.001 for TM vs. AXEF or Sily 50. Images were captured using light microscopy (100× magnification) or fluorescence filter equipped microscope (100× magnification).

We further explored the anti-NASH effects and its possible mechanisms on NASH status by treatment with TM in Huh7 cells. AXEF showed the protective effects of NASH by decreases in intracellular fat accumulations by evidence of Oil Red O staining (Positive signals for red color, Figure 5A) as well as cell death signals including caspase 3/7 activity, BAX, BcL-xL, and BcL-2 in cellular protein levels compared with the TM group (*p* < 0.01 or 0.001 for Figure 5B–E,H–J).

**Figure 6:**
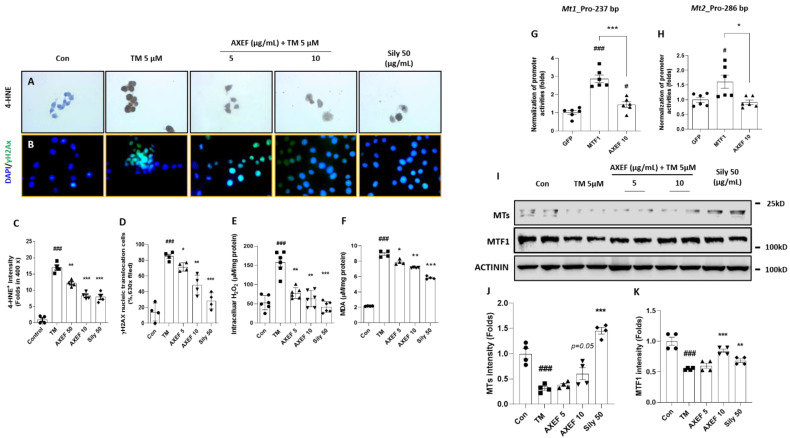
Effects of AXEF on the ER stress-induced cellular oxidation via regulation of MTs. Immunohistochemistry analysis against (**A**) 4-HNE and (**B**) immunofluorescence of γH2AX. Quantitative analysis of (**C**) 4-HNE positive signals and (**D**) γH2AX. Intracellular levels of (**E**) ROS and (**F**) MDA levels. Luciferase reporter assays for (**G**) MT1 and (**H**) MT2 promoters were performed in HEK293 cells that were transfected with respective promoter constructs with vector GFP, MTF1, or AXEF 10 µg/mL. MTF1 was used as positive control. (**I**) Western blot analysis of MTs and MTF1 from Huh7 cell protein lysate. (**J**,**K**) Quantitative analysis of MTs and MTF1 protein intensity. Data are expressed as the mean ± SD (*n* = 4 for IHC analysis and IF analysis; *n* = 6–8 for each group in intracellular H_2_O_2_ levels, MDA, *n* = 6 for gene promoter reporter assay). # *p* < 0.05 for the Con vs. TM, * *p* < 0.05 for TM vs. AXEF or Sily 50 in Figure 6C–F. # *p* < 0.05 and ### *p* < 0.001 for GFP vs. MTF1 or AXEF 10, * *p* < 0.05; ** *p* < 0.01; *** *p* < 0.001 for GFP vs. AXEF 10 in Figure 6G,H, respectively. Images were captured using light microscopy (400× magnification) or fluorescence filter equipped microscopy (630× magnification).

Regarding the cellular oxidation, TM treatment considerably increased the 4-HNE and γH2Ax signals (*p* < 0.001 for Figure 6A–D). Coinciding with these results, cellular H_2_O_2_ and MDA levels were also significantly higher, 3.1- and 4.2-fold that of the control group (*p* < 0.001 for Figure 6E,F). The above alterations were significantly normalized by AXEF compared with the TM group (*p* < 0.05, 0.01, or 0.001 for Figure 6C–F) and the antioxidant effects were also remarkably prolonged for regulation of intracellular H_2_O_2_ and MDA levels with statistical significance (*p* < 0.05 or 0.01 for Figure 6E,F).

We used Western blot analysis to explore potent hepatic antioxidants, especially MT with its transcriptional factor MTF1. The cellular protein levels of hepatic MT and MTF1 were significantly deterred by the TM treatment and these alterations were notably recovered by the pre-treatment with AXEF (especially 10 µg/mL, *p* < 0.05 or *p* = 0.05 for Figure 6I–K).

To confirm whether AXEF directly implicates MTs’ gene expression, we further explored luciferase reporter assays using proximal promoters of both mouse Mt1 and Mt2 genes in HEK 293T cells. The MT1 promoters were activated by AXEF, but the MT2 promoter was not altered by AXEF. We used a MTF1 plasmid as a positive control in the gene promoter luciferase assay (*p* < 0.05 for Figure 6H,I).

The pre-treatment with silymarin (50 µg/mL) also showed the attenuations of ER stress-provoked intracellular oxidations and the improvement of the protein levels of MT and MTF1, respectively.

## 4. Discussion

We investigated the pharmacological properties of AXEF and its underlying actions by focusing on the antioxidant activities on hepatic ER stress-induced NASH using a TM injection mice model. Regarding hepatic ER stress, AXEF worked to attenuate ER stress-related molecules by evidence of the qPCR or Western blot analysis in both in vivo and in vitro models, respectively (Figure 1A–D, Figure 5E).

PERK signaling pathways on the ER stress status, increases in p-PERK activates eIF2α, which can mediate the nucleic translocation of ATF4 for further pathological states including apoptosis, oxidative stress, and redox homeostasis [40,41]. During hepatic ER stress by TM injection, the hepatic tissue suffered from fatty liver conditions by hepatocyte ballooning, an increase in TG levels, and up-regulation of lipogenesis genes, which are the main pathophysiological features of NASH (Figure 1E–J and Supplementary Figure S2). Accompanying the above alterations, liver injury was also observed by increases in serum biochemistries (Figure 1H–J). Both the gene expression and hepatic protein levels were well supported by these pathological statuses (Figure 2A–D). According to our intention, pre-administration with AXEF worked drastically to normalize these alterations.

It is well documented that TM is a bacterial nucleoside antibiotic, which can block *N*-linked glycoproteins and allow unfolded or misfolded proteins to deposit in the ER lumen by activation of the activates’ unfolded protein response (UPR) [42]. According to the above actions, hepatic ER stress by TM injection mice models [43,44] have been broadly used to study and understand the specific mechanism of acute pharmacologic ER stress mediated liver metabolism, oxidative stress, redox homeostasis, chaperone, and cell death, respectively [45,46,47].

In the present study, we explored the pharmacological properties of AXEF by focusing on oxidative stress [11,21]. Previous studies have documented well that hepatic ER stress and oxidative stress are deeply implicated, and many study groups have tried to understand the pathophysiological status to develop therapeutics for liver diseases [48,49,50]. AXEF, as we expected, strongly protected against oxidative stress in the liver tissue during ER stress by relieving the of tissue ROS (DHE and DCF-DA), H_2_O_2_ levels, MDA contents, and 4-HNE signals (Figure 4A–E). Simultaneously, AXEF recovered the deteriorations of endogenous antioxidant and total GSH contents (Figure 4F). The cellular oxidation of Huh7 cells was also observed in the TM treatment group but was mostly resolved by the AXEF pre-treatment (Figure 6A–E).

According to previous studies, AX was applicated to treat liver tissue oxidation with various extraction types using chronic chemical stimulus models via the enhancement of antioxidant capacities [32,51]. In addition, accumulated evidence reported the possibilities of AXEF on the hepatic ER stress-evoked oxidative stress conditions well [30,31,33]. Previously, chronic liver fibrosis models, such as dimethylnitrosamin (DMN, also known as N-Nitrosodimethylamine)-induced and bile duct ligation models, elevated hepatic oxidative stress, but AXEF resolved them [30,31]. Another study also showed that haptic steatosis with mild inflammatory conditions due to eight weeks of being fed a high fat diet (HFD) was improved by AXEF administrations [33]. Although these studies revealed the pharmacological properties of AXEF as a potent antioxidant, there is still a lack of proof regarding its underlying mechanisms. Additionally, there is no study on hepatic ER stress and AX (even in other types of AX extraction) focusing on liver diseases.

As a response to oxidative stress during ER stress in hepatic tissue, the final stage of pathological status was progressed by an enhancement of cell death signals [10]. Our TUNEL assay results presented the anti-apoptosis effects of AXEF in the liver tissue, which were supported by pro-and anti-apoptotic proteins (Figure 4A–G). Similar to the in vivo condition, the in vitro experiment also displayed the protective effects of AXEF by the prevention of cell death signals (Figure 5B–E,H–J). In our cell model of hepatic ER stress, we observed that DNA oxidation happened in this model (evidenced by γH_2_Ax), and this may be deeply associated with cell death (Figure 6B–D). Furthermore, the mice hepatic liver condition of the anti-apoptotic protein, BAX, was significantly decreased by AXEF, but was not altered in the cell models (Figure 4D,E; Figure 5E,H).

In the present study, we mainly displayed the antioxidant activities of AXEF and refreshingly discovered that the hepatic MT levels, which are a target transcription of MTF1, were reduced by the TM injection condition of mice liver tissues (Figure 3G–I). Coinciding with an in vivo condition, an in vitro experiment also reenacted this pathological status well (Figure 6I–K). To validate the direct implication between MTs and AXEF physiologically, the luciferase reporter assay showed that the MT1 promoter activities were significantly increased by the AXEF treatment (Figure 6G). MTs are known for being cysteine-rich proteins, and they show hepatoprotective effects in certain pathological conditions, such as NASH and alcoholic steatohepatitis (ASH) [52,53]. During liver tissue oxidation, MTs can achieve these beneficial effects by scavenging free radicals to suppress oxidative stress, leading to the homeostasis of redox signaling, and alleviating apoptosis signals [54,55]. Contrary to a previous study, our mice model displayed hepatic MT depletion [56]. This phenomenon may be attributed to the TM injection method and duration, and it could hinder the synthesis against hepatic ER stress. Despite a steady increase in the prevalence rates of NASH, there is currently no effective therapeutic treatment. Owing to the above issue, it would be expected that many more NASH patients progress to an advanced stage of liver diseases including fibrosis, cirrhosis, and HCC [13]. Thus, it sounds urgent to develop an appropriate therapeutic.

In the current study, we provide the possibilities to use AXEF in a hepatic ER stress-induced NASH model in various ways. AXEF showed protective effects via decreases in liver enzymes and hepatic TG during ER stress. Moreover, AXEF improves hepatic tissue oxidation via the enhancement of antioxidant capacities by quenching oxidative stressors. The corresponding pharmacological actions may be linked to the enhancement of hepatic MT expression levels. These effects may be attributed to increases in GSH synthesis and prevent hepatocyte death as well.

## 5. Conclusions

Taken together our data support the possibility that supplementation with antioxidants may help to inhibit the progression of NASH. Further studies of AXEF are still needed to properly investigate the safety of this drug for its therapeutic application in clinical settings.

## Data Availability

Data is contained within the article.

## Supplementary Materials

The following are available online at https://www.mdpi.com/article/10.3390/antiox10070998/s1, Figure S1: Fraction of AXEF and its fingerprinting analysis, Figure S2: Hepatic mRNA expression levels of lipogenesis related genes, Table S1: Antibody lists, Table S2: Primer lists.
