# Supplementary material for: Ethyl Acetate Fraction of Amomum villosum var. xanthioides Attenuates Hepatic Endoplasmic Reticulum Stress-Induced Non-Alcoholic Steatohepatitis via Improvement of Antioxidant Capacities
Source: Antioxidants (Basel). 2021 Jun 23;10(7):998. doi: 10.3390/antiox10070998 (PMC8300789; doi:10.3390/antiox10070998)
Supplement: Supplementary file 1 [file antioxidants-10-00998-s001.zip › Supplementary Figure S1-Finger printing.pptx]

## Slide 1
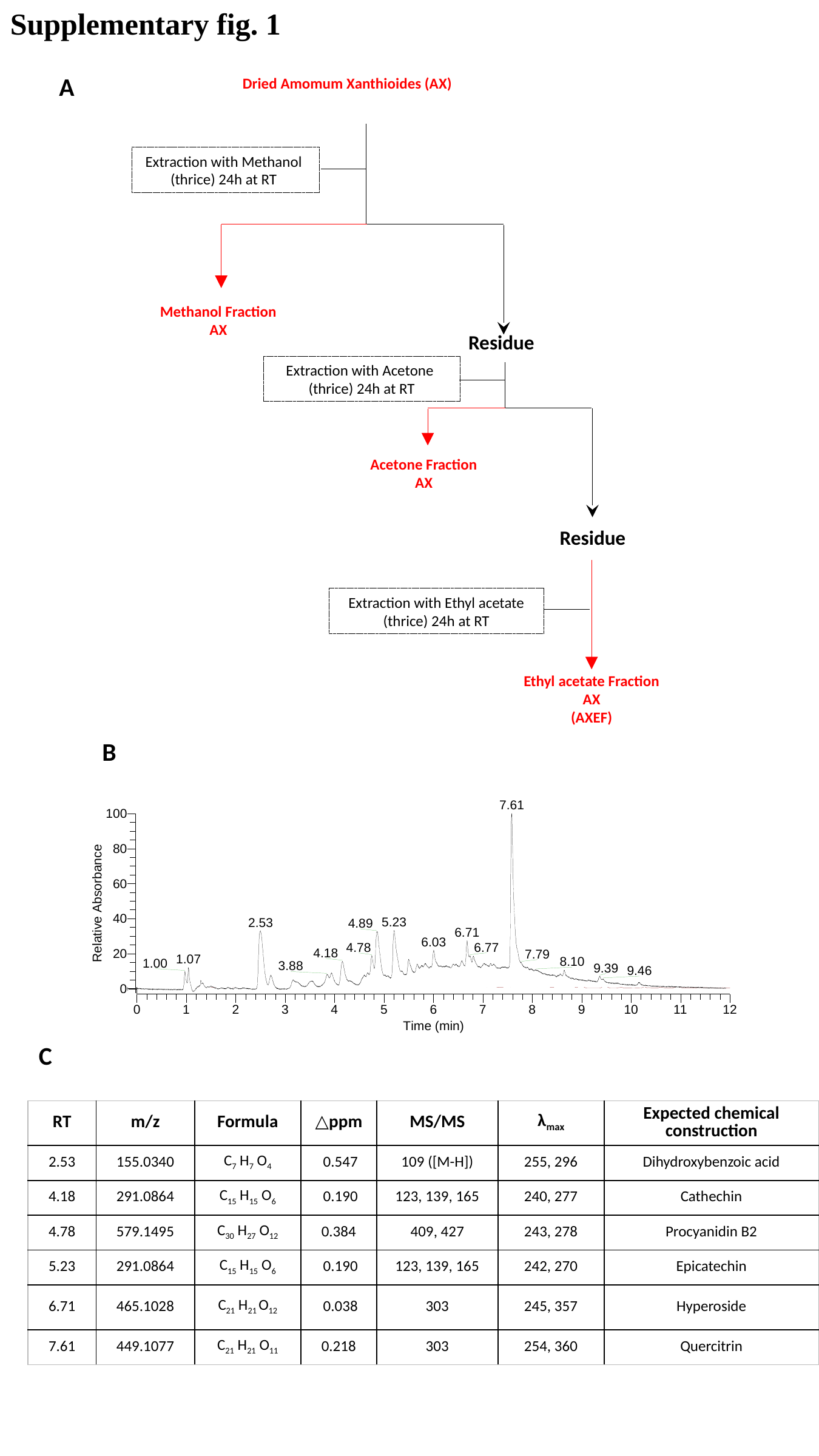

Supplementary fig. 1
A
Dried Amomum Xanthioides (AX)
Extraction with Methanol
(thrice) 24h at RT
Methanol Fraction
AX
Residue
Extraction with Acetone
(thrice) 24h at RT
Acetone Fraction
AX
Residue
Extraction with Ethyl acetate
(thrice) 24h at RT
Ethyl acetate Fraction
AX
(AXEF)
B
C
| RT | m/z | Formula | △ppm | MS/MS | λmax | Expected chemical construction |
| --- | --- | --- | --- | --- | --- | --- |
| 2.53 | 155.0340 | C7 H7 O4 | 0.547 | 109 ([M-H]) | 255, 296 | Dihydroxybenzoic acid |
| 4.18 | 291.0864 | C15 H15 O6 | 0.190 | 123, 139, 165 | 240, 277 | Cathechin |
| 4.78 | 579.1495 | C30 H27 O12 | 0.384 | 409, 427 | 243, 278 | Procyanidin B2 |
| 5.23 | 291.0864 | C15 H15 O6 | 0.190 | 123, 139, 165 | 242, 270 | Epicatechin |
| 6.71 | 465.1028 | C21 H21 O12 | 0.038 | 303 | 245, 357 | Hyperoside |
| 7.61 | 449.1077 | C21 H21 O11 | 0.218 | 303 | 254, 360 | Quercitrin |
