# Supplementary material for: Ethyl Acetate Fraction of Amomum villosum var. xanthioides Attenuates Hepatic Endoplasmic Reticulum Stress-Induced Non-Alcoholic Steatohepatitis via Improvement of Antioxidant Capacities
Source: Antioxidants (Basel). 2021 Jun 23;10(7):998. doi: 10.3390/antiox10070998 (PMC8300789; doi:10.3390/antiox10070998)
Supplement: Supplementary file 1 [file antioxidants-10-00998-s001.zip › Supplementary information.pdf]

Supplementary information

Supplementary figure 1.

A

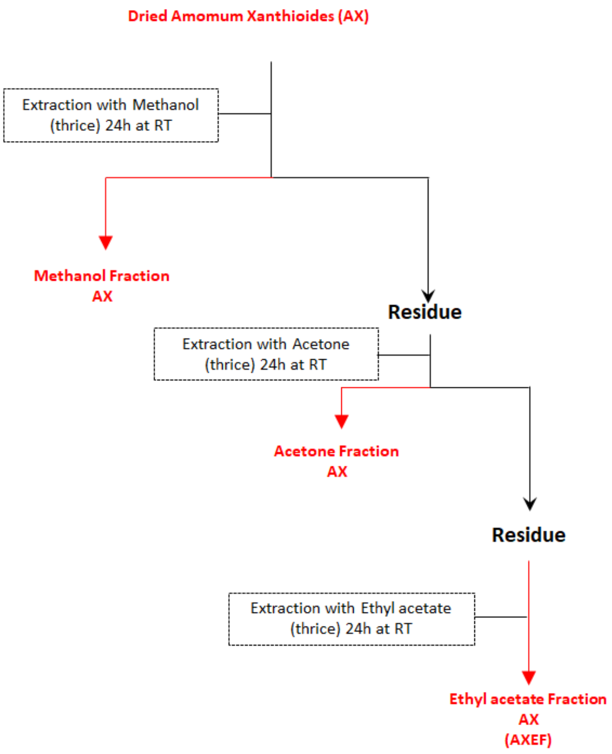

B

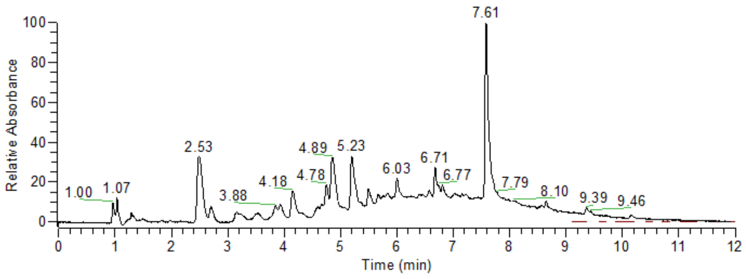

C

RT	m/z	Formula	△ppm	MS/MS	λ_{max}	Expected chemical construction
2.53	155.0340	$C_7H_7O_4$	0.547	109 ([M-H])	255, 296	Dihydroxybenzoic acid
4.18	291.0864	$C_{15}H_{15}O_6$	0.190	123, 139, 165	240, 277	Cathechin
4.78	579.1495	$C_{30}H_{27}O_{12}$	0.384	409, 427	243, 278	Procyanidin B2
5.23	291.0864	$C_{15}H_{15}O_6$	0.190	123, 139, 165	242, 270	Epicatechin
6.71	465.1028	$C_{21}H_{21}O_{12}$	0.038	303	245, 357	Hyperoside
7.61	449.1077	$C_{21}H_{21}O_{11}$	0.218	303	254, 360	Quercitrin

Supplementary figure 1. Fraction of AXEF and its fingerprinting analysis. (A) Diagram of obtaining AXEF according to the fractionation relying on the solvent polarity. From methanol extraction to ethyl acetate fraction (AXEF). (B) Two dimensional histogram of AXEF analysis by adaption of ultra-high-performance liquid chromatography-tandem mass spectrometry. (C) Major chemical constructions from AXEF.

Supplementary figure 2.

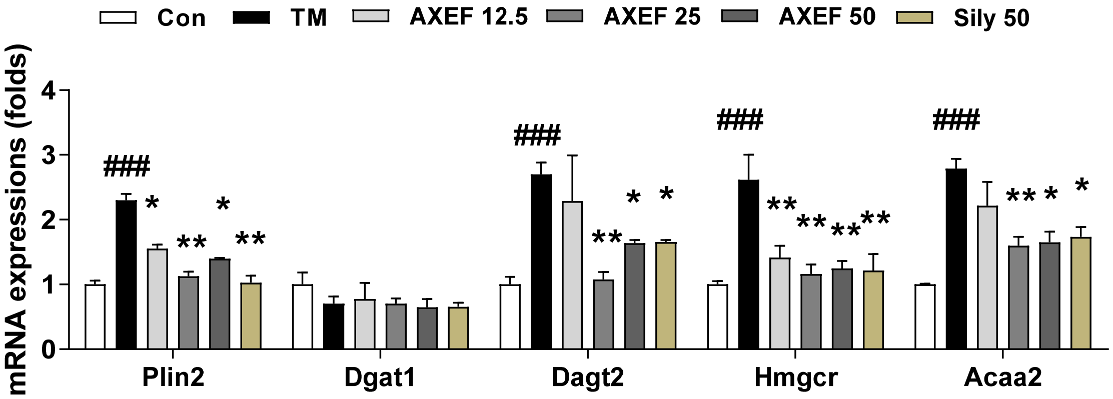

Supplementary figure 2. Hepatic mRNA expression levels of lipogenesis related genes. Data were expressed mean ± SD (n= 6-8 for each group). # P < 0.05 for the Con vs. TM, **P* < 0.05 for TM vs. AXEF or Sily 50. Acca2; Acetyl-CoA Acyltransferase 2, Dgat; Diglyceride acyltransferase, Plin2; Perplin2, Hmgcr; 3-hydroxy-3-methylglutaryl coenzyme A reductase,

Supplementary table 1. Antibody lists

Name	Host	Application	Titer	Manufacture and Lot. No
PERK	Rb	WB	1:1000	CST, PERK (C33E10) Rabbit mAb #3192
p-PERK	Rb	WB	1:500	p-PERK-T982, Custom rabbit monoclonal rIgG; LLY-71, clone 1-6 Lot #BE02734-048
eIF2α	Rb	WB	1:1000	CST, eIF2α (D7D3) XP® Rabbit mAb, #5324
p-eIF2α	Rb	WB	1:500	CST, Phospho-eIF2α (Ser51) (D9G8) XP® Rabbit mAb, #3398
Bax	Ms	WB	1:1000	Thermo Fischers, Bax Monoclonal Antibody (6A7), MA5-14003
Bcl-XL	Ms	WB	1:1000	Thermo Fischers, Bcl-xL Monoclonal Antibody (7D9), MA5-11950
Bcl2	Ms	WB	1:1000	Thermo Fischers, Bcl-2 Monoclonal Antibody (100/D5), MA5-11757
MTs	Ms	WB	1:500	Enzo Life, ADI-SPA-550-D
MTF1	Rb	WB	1:500	Fishers, PIPA555945
Actinin	Rb	WB	1:3000	Santa Cruz Biotechnology, sc-7454-R, alpha-Actinin (C-20)-R
4-HNE	Ms	IHC	1:200	R&D, MAB3249,
γH2AX	Rb	IF	1:200	CST, Phospho-Histone H2A.X (Ser139/Tyr142) Antibody, #5438

PERK; protein kinase R (PKR)-like endoplasmic reticulum kinase, eIF2α; Eukaryotic translation initiation factor 2A, Bcl2; Bcl-2 (B-cell lymphoma, Bax; BCL2 Associated X, Apoptosis Regulator, Bcl-XL; B-cell lymphoma-extra-large, MTs; Metallothioneins, MTF1; Metal regulatory transcription factor 1, 4-HNE; 4-hydroxy-2-nonenal, γH2AX; phoshpor- H2A histone family member X

Supplementary table 2. Primer lists

Gene name	Sequence (5' to 3')	Application
Grp78/Bip	5′-GTG TGT GAG ACC AGA ACC GT-3′	qPCR
	5′-GCA GTC AGG CAG GAG TCT TA-3′	
Ddit3/Chop	5′-TCT TGA GCC TAA CAC GTC GAT-3′	
	5′-CCA GGT TCT CTC TCC TCA GGT-3′	
Tnfa	5'- TGCCTATGTCTCAGCCTCTTC-3'	
	5'- GGAGGCCATTTGGGAACT-3'	
Il6	5'- CAA AGC CAG AGT CCT TCA GAG-3'	
	5'- GAG CAT TGG AAA TTG GGG TA-3'	
Il1b	5'- TGT GAA ATG CCA CCT TTT GA-3'	
	5'- GGT CAA AGG TTT GGA AGC AG-3'	
Il10	5'- CAG AGC CAC ATG CTC CTA GA-3'	
	5'- GCT TGG CAA CCC AAG TAA CC-3'	
Plin2	5'- TCCACTGTCCACCTGATTGA-3'	
	5-TGGCATGTAGTCTGGAGCTG-3'	
Dgat1	5′-TCC GCC TCT GGG CAT TC-3'	
	5′-AGA ATC GGC CCA CAA TCC A-3'	
Dgat2	5′-CAAGAAAGGTGGCAGGAGAT-3'	
	5′-GTGGTCAGCAGGTTGTGTGT-3'	
Hmgcr	5'- ATCCTGACGATAACGCGGTG-3'	
	5'- AAGAGGCCAGCAATACCCAG-3'	
Acaa2	5'- CCCTGCTACGAGGTGTGTTC-3'	
	5'- ACATTGCCCACGATGACACT-3'	
Mt1-Pro	5′-AAT TGC TAG CGG AAA GCA CTA TAG GGA CAT GA-3'	Gene promoter reporter assay
	5′-AAT TCT CGA GTC TTT ATA GTC GTT GGA CGA GT-3′	
Mt2-Pro	5′-AAT TGC TAG CGG ATC GCA GAC CCT TTG C-3′	
	5′-AAT TCT CGA GCC TTT ATA GCG GAG AGT ATT GG-3′	
β-actin	55′- GGC ACCACACCTTCTACAATGA-3′	qPCR
	5′- ATCTTTTCACGGTTGGCCTTAG-3′	

Acca2; Acetyl-CoA Acyltransferase 2, Ddit3; DNA damage-inducible transcript 3, Chop; C/EBP homologous protein, Dgat; Diglyceride acyltransferase, Plin2; Perplin2, Hmgcr; 3-hydroxy-3-methylglutaryl coenzyme A reductase, Tnf; tumor necrosis factor, Il; interleukin
