# Supplementary figures and images for: Ethyl Acetate Fraction of Amomum villosum var. xanthioides Attenuates Hepatic Endoplasmic Reticulum Stress-Induced Non-Alcoholic Steatohepatitis via Improvement of Antioxidant Capacities
Source: Antioxidants (Basel). 2021 Jun 23;10(7):998. doi: 10.3390/antiox10070998 (PMC8300789; doi:10.3390/antiox10070998)

## Slide 1
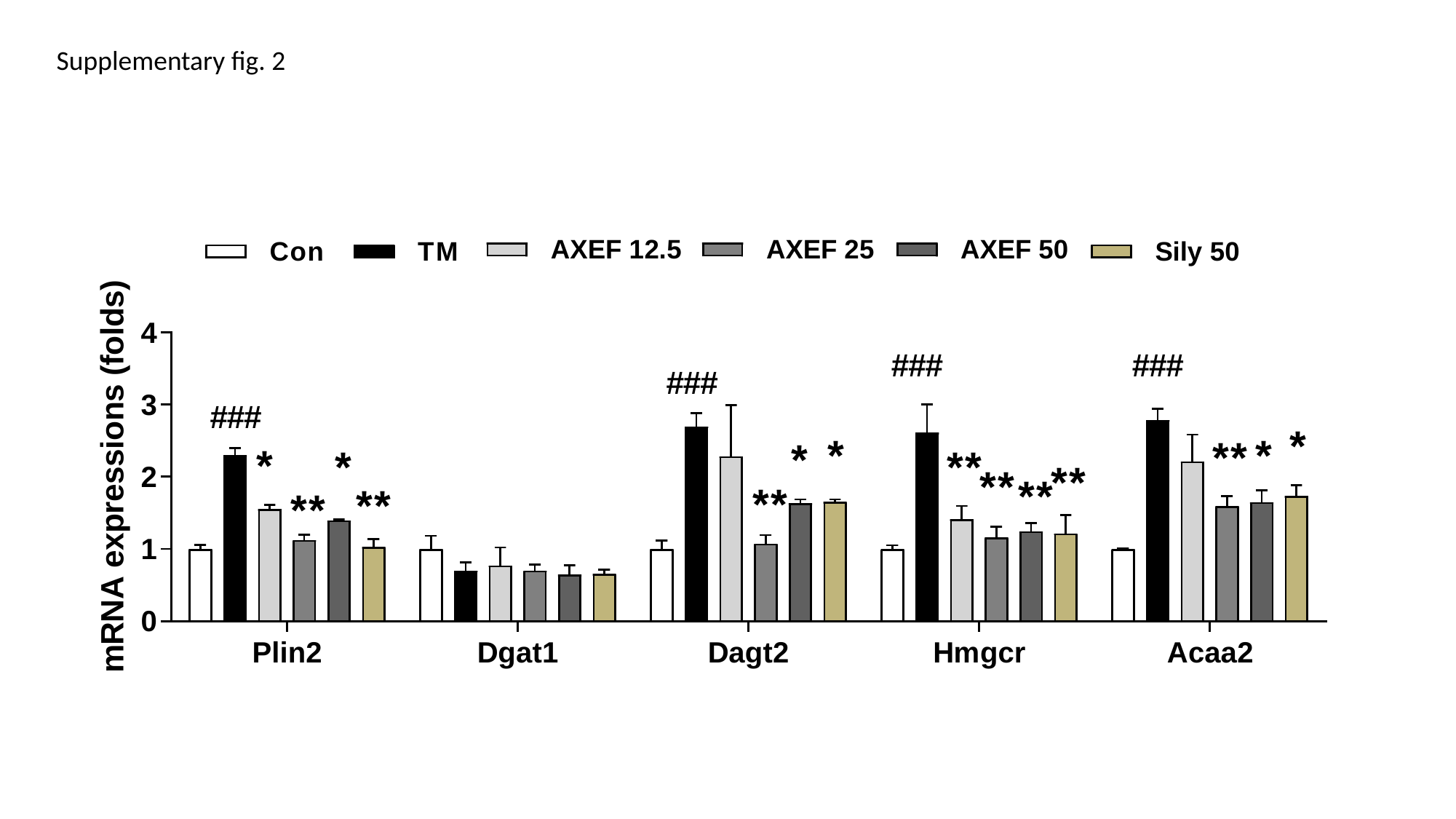

Supplementary fig. 2

Supplement: Supplementary file 1 [file antioxidants-10-00998-s001.zip › Supplementary Figure S2-Gene expression analysis.pptx]
